# Visualization of X chromosome reactivation in mouse primordial germ cells *in vivo*

**DOI:** 10.1242/bio.058602

**Published:** 2021-04-29

**Authors:** Yoshikazu Haramoto, Mino Sakata, Shin Kobayashi

**Affiliations:** 1 Cellular and Molecular Biotechnology Research Institute, National Institute of Advanced Industrial Science and Technology (AIST), Central 6, 1-1-1 Higashi, Tsukuba, Ibaraki 305-8566, Japan; 2 Cellular and Molecular Biotechnology Research Institute, National Institute of Advanced Industrial Science and Technology (AIST), 2-4-7 Aomi, Koutou-ku, Tokyo 135-0064, Japan; 3 Department of Epigenetics, Medical Research Institute, Tokyo Medical and Dental University (TMDU), 1-5-45 Yushima, Bunkyo-ku, Tokyo 113-8510, Japan

**Keywords:** X chromosome reactivation (XCR), X chromosome inactivation (XCI), Primordial germ cell (PGC), Momiji system, Reprogramming, Imaging

## Abstract

X chromosome inactivation (XCI), determined during development, remains stable after embryonic cell divisions. However, primordial germ cells (PGCs) are exceptions in that XCI is reprogrammed and inactivated X chromosomes are reactivated. Although interactions between PGCs and somatic cells are thought to be important for PGC development, little is known about them. Here, we performed imaging of X chromosome reactivation (XCR) using the ‘Momiji’ mouse system, which can monitor the X chromosome's inactive and active states using two color fluorescence reporter genes, and investigated whether interactions would affect XCR in PGCs. Based on their expression levels, we found that XCR of the *Pgk1* locus began at embryonic day (E)10.5 and was almost complete by E13.5. During this period, PGCs became distributed uniformly in the genital ridge, proliferated, and formed clusters; XCR progressed accordingly. In addition, XCR of the *Pgk1* locus preceded that of the *Hprt* locus, indicating that the timing of epigenetic memory erasure varied according to the locus of each of these X-linked genes. Our results indicate that XCR proceeds along with the proliferation of PGCs clustered within the genital ridge.

This article has an associated First Person interview with the first author of the paper.

## Abstract (precis)

**Summary:** Imaging of mouse X chromosome activities reveals that they undergo reprogramming and reactivation while the primordial germ cells form clusters and proliferate in the genital ridge.

## INTRODUCTION

X chromosome inactivation (XCI) is an epigenetic mechanism characteristic of Eutheria that equalizes the expression of X-linked genes between male and female mammals by inactivating one of the two X chromosomes in females. This epigenetic silencing is established during early embryonic development and maintained stably thereafter through embryonic cell divisions. It is generally believed that XCI always occurs in differentiated cells. If this mechanism fails, it leads to death during early development, so it is an important gene regulatory mechanism that requires strict control (Marahrens et al., 1997). In the mouse – the most frequently studied animal model – whether the paternally or maternally derived X chromosome is inactivated is known to change dynamically depending on developmental stages and tissues (Augui et al., 2011; Jeon et al., 2012; Kobayashi, 2017; Pasque and Plath, 2015).

In the development of female mammals, there are two exceptional cases of X chromosome reactivation (XCR) in which the epigenetic memories of XCI are erased and both X chromosomes become activated. One occurs in the inner cell mass (ICM) of the blastocyst (destined to form the fetus) and/or the epiblast during peri-implantation stages, and the second occurs in primordial germ cells (PGCs) destined to form oocytes. A common property of these cells exhibiting XCR is that they are in a highly undifferentiated state (Ohhata and Wutz, 2013; Pasque and Plath, 2015). In particular, PGCs undergo epigenetic reprogramming and serve as totipotent precursors for all cell types. This is an important step in transmitting genetic information to the next generation.

In mice, dozens of PGCs appear in the extraembryonic mesoderm on day 7 of embryonic development, and then migrate from the proximal epiblast and reach the genital ridge (GR) at embryonic day (E)10.5 to undergo maturation (Ohinata et al., 2005). In this process, the resetting of epigenetic information begins, such as genome-wide DNA demethylation and dynamic changes to histone modifications, leading to the reacquisition of pluripotency and the initiation of active PGC proliferation reviewed in (Molyneaux and Wylie, 2004; Saitou et al., 2012; Saitou and Yamaji, 2012). The PGCs start proliferation at E9.5 onward, and the numbers increase explosively in the GR. XCR is thought to progress at the same time as genome-wide reprogramming. The reconstitution of female germ cell development *in vitro* has shown that the interaction between PGCs and somatic cells is important for PGC maturation and reprogramming (Hayashi et al., 2012), but the details of these interactions are unknown. Thus, it is not well understood whether there are special tissues that induce XCR in the migration route of PGCs, or whether XCR occurs in specific locations of the GR. To address these questions, we have developed genetically modified mice (named the Momiji mouse system) that can be used to monitor X chromosome status (Kobayashi et al., 2016). In this system, red and green fluorescent protein reporter genes are inserted into each of the two X chromosomes, and epigenetic differences between active and inactive X chromosomes can be detected at the single-cell level as differences in fluorescent protein expression. Here, using this system, we followed PGCs in developing mouse embryos and observed the changes in X chromosome status.

At the initiation of XCI, it is considered that the entire X chromosome is not inactivated simultaneously, and that there are positional effects in the X chromosome: i.e., there are differences in the timing of inactivation in different X-linked genes. By using the Momiji system with reporter genes inserted at two different positions on the X chromosome (*Pgk1* and *Hprt* loci), we found previously that the *Hprt* locus was inactivated earlier than the *Pgk1* locus in the initiation of random XCI (rXCI) that occurs immediately after implantation (∼E6.5), so that the timing of initiation of rXCI is different for each X-linked gene (Kobayashi et al., 2016). Here, we analyzed whether similar positional effects lead to differences in the timing of XCR in PGCs, which is a reprogramming event erasing rXCI memories.

## RESULTS

We detected differences in the epigenetic state of two X chromosomes based on the color of two different fluorescent reporter proteins: enhanced green fluorescent protein (eGFP) and mCherry. If both X chromosomes are activated, the green and red proteins are both activated as well, and a yellow signal is detected. If one of them is inactivated, a monochromatic red or green signal is detected (Fig. S1A). In this system, two different insertion sites of reporter genes enable us to detect the positional effects of epigenetic regulation in XCR (Fig. S1B: *Pgk1* and *Hprt* loci). Based on this scheme, we distinguished which cell showed XCR at single-cell resolution during PGC migration and subsequent proliferation in the GR.

### XCR starts nonsynchronously and the timing varies between cells

**Fig. 1.**
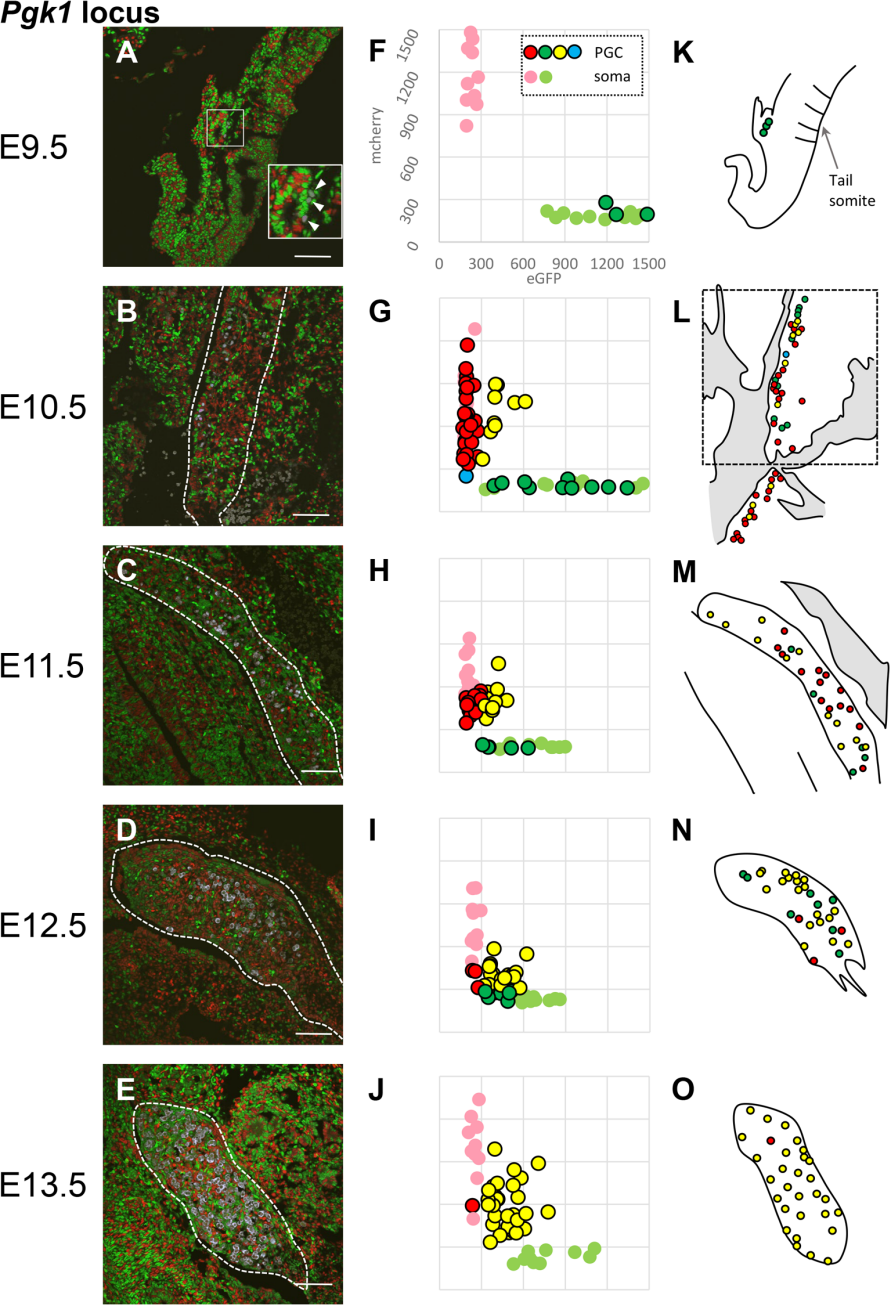
**Observation of X chromosome reactivation of the *Pgk1* locus in PGCs at each developmental stage.** (A–E) The merged image of eGFP (green), mCherry (red), and immunostaining with PGC markers (Oct3/4 for E9.5–E11.5, and Mvh for E12.5 and E13.5) (white) is shown. The position of the GR is indicated by a dotted line. A twofold magnified view of the boxed region in A is shown in the inset. White arrowheads in the inset indicate PGCs. The boxed region in L indicates the area shown in B. Scale bars: 100 µm. (F–J) Detection of XCR by quantitative analysis of fluorescence intensity in PGCs. Each spot corresponds to one cell. Ten single positive somatic cells in light red or light green are plotted. PGC marker-positive cells are shown as circles outlined in black, and cells with red and green signal intensities both ≥300 units are shown as yellow. Light blue spots show cells with no signal (both red and green signal intensities <300). Prior to E10.5, all PGCs in a slide are plotted. Because the numbers of PGCs increase massively after E11.5, the number of PGCs in these plots was limited to a maximum of 30. (K–O) Schematic representation of the XCI (red or green) or XCR (yellow) of PGCs located in the genital ridge. The positions of PGCs whose signal intensities were measured in F–J are shown in these images. Developmental stages are indicated on the left side of the panels. For each stage, at least three embryos were analyzed, and a representative one is shown here.

It is generally accepted that there is an interaction between the GR and PGCs, so we investigated whether this would affect XCR *in vivo*. We focused on whether there are spatial characteristics about the proliferation of PGCs and the progression of XCR. Based on the knowledge of PGC migration toward the GR, it seemed likely that the location and patterns of XCR among PGCs in the GR would show characteristic features depending on the timing of arrival. We analyzed the proliferation of PGCs and the progression of XCR in *Pgk1* during embryogenesis (Fig. 1; Fig. S2). We also measured the signal intensity of randomly selected PGCs (Fig. 1F–J) and have schematically illustrated the position of the measured PGCs in the GR (Fig. 1K–O). In this analysis, we identified PGCs by immunostaining using Oct3/4 and/or Mvh as PGC markers and measured the fluorescence signals of each cell. There were very few PGCs migrating toward the GR at E9.5, and XCR had not yet occurred (Fig. 1A, F). From E10.5 when the PGCs reached the GR, as the number of PGCs increased, the numbers that underwent XCR also increased gradually (Fig. 1B–D, G–I; Fig. 3A), and by E13.5, most of the PGCs had completed XCR (Fig. 1E, J; Fig. 3A). PGC proliferation and the progression of XCR did not occur at specific locations within the GR, but instead occurred in a scattered manner (Fig. 1A–E, K–O). There was a tendency for fluorescence signals from active X chromosomes to decrease first (Fig. 1G, H), followed by XCR (Fig. 1H, I); finally, the amount of fluorescent protein expressed from both alleles increased gradually (Fig. 1J). We anticipate that the relationship between the amounts of transcripts from the two X chromosomes will be clarified by RNA sequencing analysis.

### XCR progresses as PGCs multiply in clusters in the GR

**Fig. 2.**
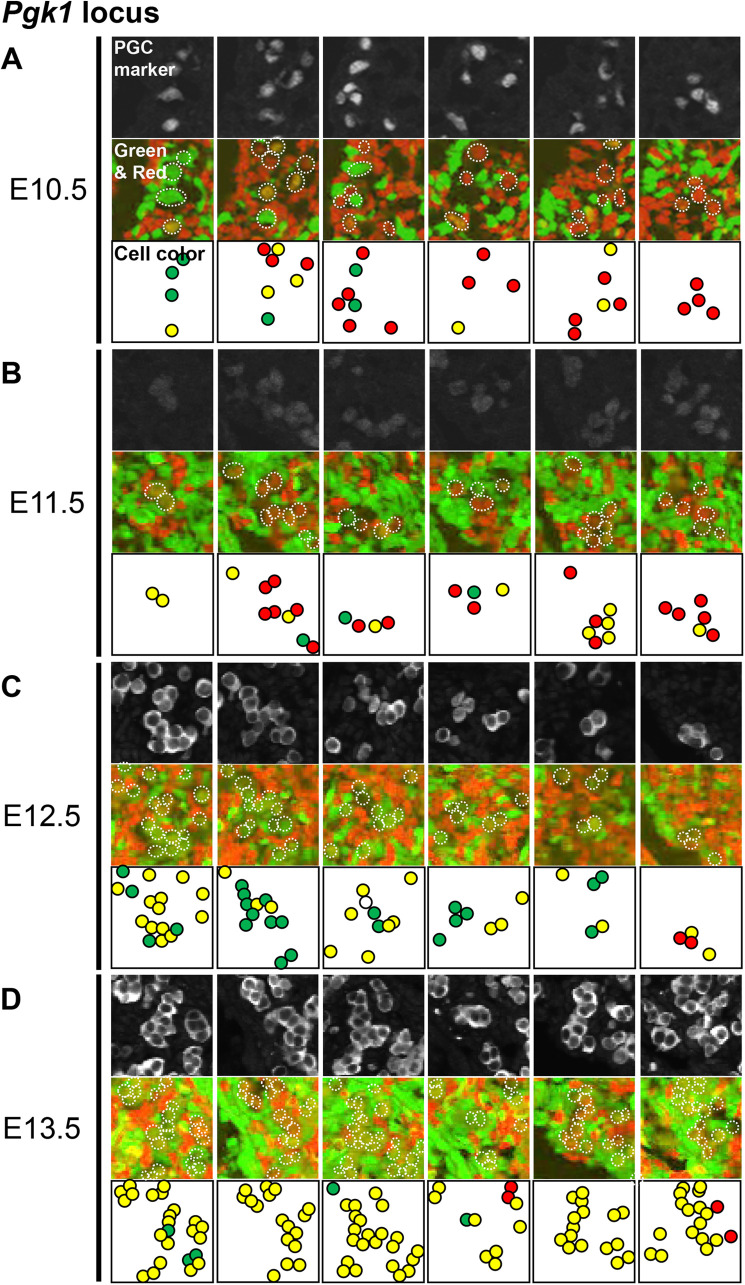
**XCR of PGCs proceeds as they proliferate by forming clusters.** Selected immunostaining images for PGC markers (Oct4 for E10.5 and E11.5, and Mvh for E12.5 and E13.5) (top), mCherry and eGFP fluorescence merged images (middle), and schematic illustrations of PGC classified by color based on fluorescence quantification are shown (bottom). PGCs with mCherry or eGFP signal intensities >300 are shown as red or green, respectively, and those with both red and green signal intensities >300 are shown as yellow. The cells in which XCR had just begun are shown as orange or yellowish green (middle). According to the judgment criteria, they are shown in yellow for convenience (bottom). The corresponding PGCs are indicated by dotted circles in the middle panels. (A) E10.5, (B) E11.5, (C) E12.5, and (D) E13.5.

**Fig. 3.**
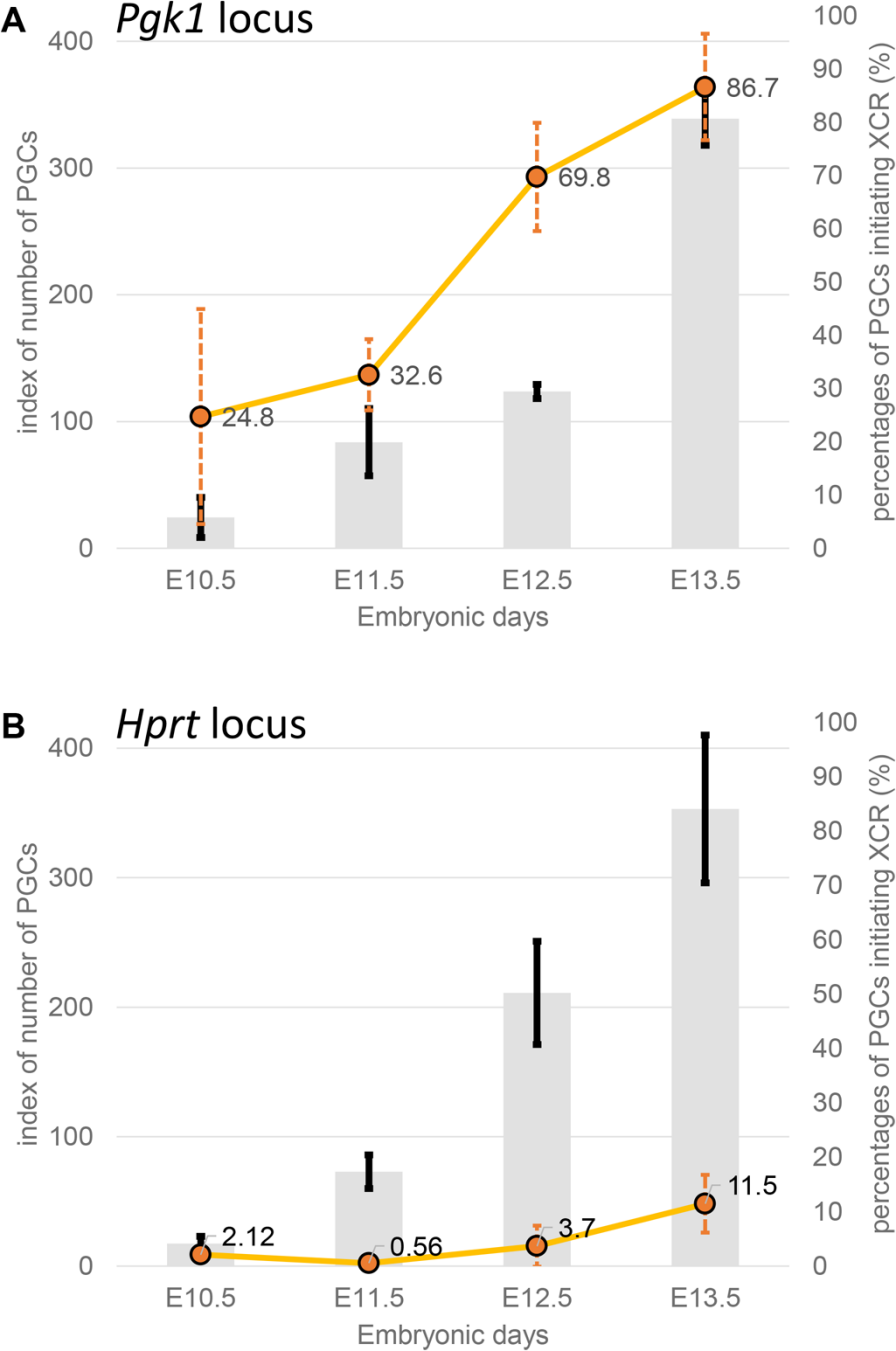
**Changes in the number of PGCs and the XCR rate during development based on the *Pgk1* and *Hprt* loci.** The bar graph represents an index of the number of PGCs (gray bars), and the line graph represents the XCR rate (orange dots and lines), calculated based on the Momiji mouse system with reporters inserted into the *Pgk1* locus (A) or *Hprt* locus (B) (see Supplementary Tables S1 and S2, and Materials and Methods for the definition of provisional index). The data are shown as the mean±standard deviation, *n*=3 (exception: E10.5 data for the *Hprt* locus are based on *n*=2).

The PGCs that migrated and reached the GR were not spatially localized and were scattered at E10.5 (Fig. 2A). These PGCs started proliferating individually and undergoing XCR at E11.5 (Fig. 2B). From E12.5 to E13.5, the proliferation of PGCs within the GR followed a characteristic pattern. Although we did not trace the proliferation of single cells using time-lapse imaging, a single PGC seemed to form a core and proliferate, forming a cluster (Fig. 2C,D). Each such cluster was located uniformly in the GR, and the PGCs in each of them underwent XCR independently, not synchronously (Fig. 2C,D). During embryogenesis, the numbers of PGCs forming each cluster increased, as did the proportions of PGCs undergoing XCR in each cluster (Fig. 2A–D). Thus, XCR progressed simultaneously with proliferation, and most of the PGCs were reactivated between E12.5 and E13.5 when we focused on the *Pgk1* locus (Fig. 3A).

### Difference in the timing of XCR depending on X chromosomal locus

Next, we analyzed XCR during PGC development using mice with the *Hprt* insertional site. Reporter analysis at two different insertional positions revealed that the timing of reactivation differed between the *Pgk1* and *Hprt* loci. At the *Pgk1* locus, XCR started at ∼E10.5 and was completed at E13.5 in almost all PGCs (Fig. 1, Fig. 3A; Fig. S2, Table S1). By contrast, at the *Hprt* locus, XCR started at E11.5, but even at E13.5, XCR was only observed in about 10% of PGCs and was still not complete (Fig. 3B; Fig. S3, S4, Table S2), indicating that the timing of reactivation depends on the X chromosome locus. For the *Hprt* locus in the Momiji system, we could not determine exactly when XCR was completed. This was because the fluorescence intensity weakened in PGCs after E13.5, most probably because of the low level of transcriptional activity, as reported (Lebedeva et al., 2018). In neonatal gonads, more than 90% of PGCs had undergone XCR, even at the *Hprt* locus, confirming that XCR had been completed during oocyte maturation and that our reporter system worked well (Table S2). Using these Momiji mice, we previously analyzed the initiation of rXCI in the ICM after implantation and found that the *Hprt* locus started rXCI before the *Pgk1* locus (Kobayashi et al., 2016). Thus, the *Hprt* locus completes rXCI quickly and XCR slowly, while this timing is reversed for the *Pgk1* locus.

**Fig. 4.**
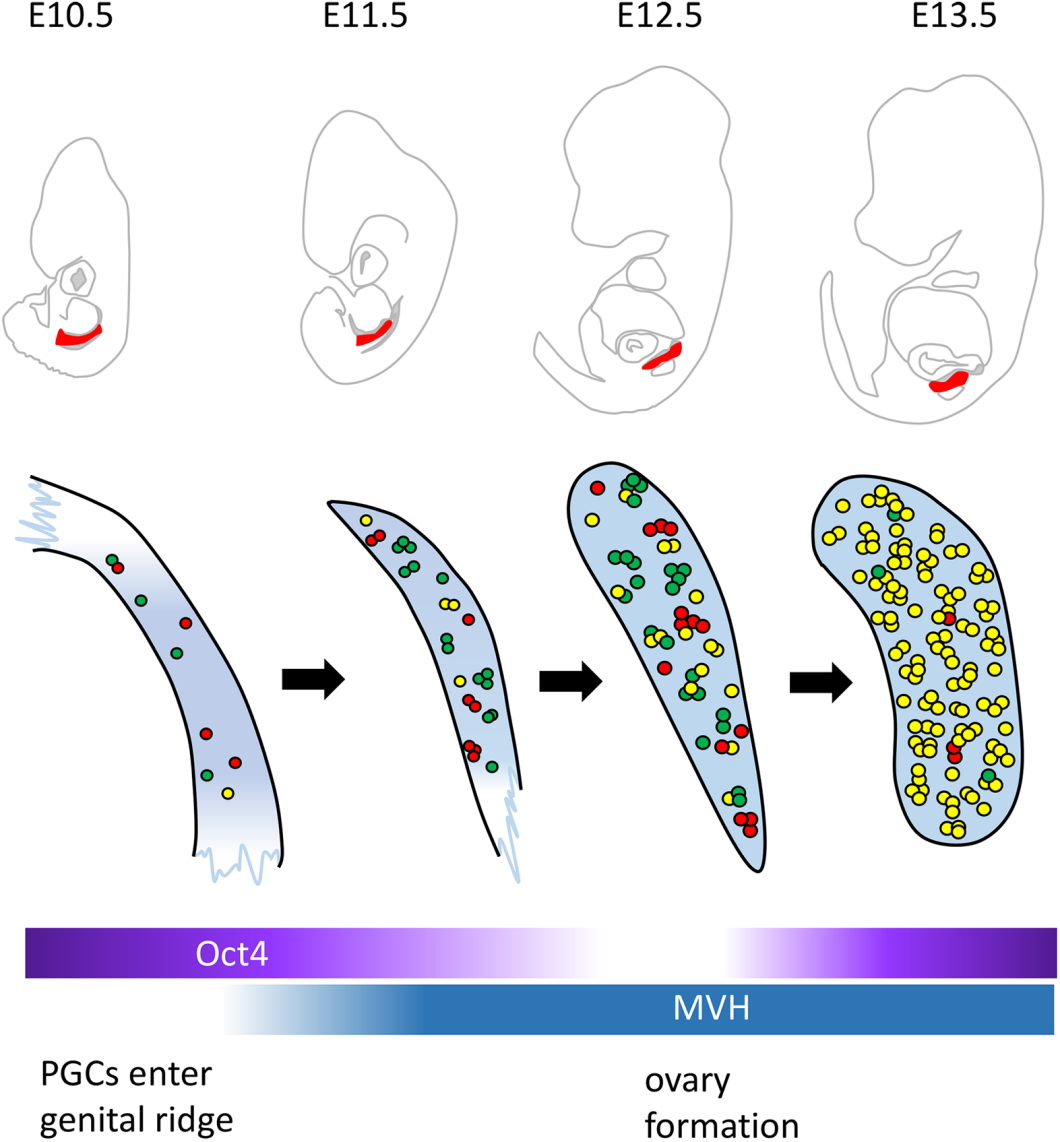
**Schematic diagram of PGC proliferation and X chromosome reactivation during embryogenesis.** PGCs in the genital ridge are evenly distributed. Each PGC proliferates and forms clusters. Reactivation at the *Pgk1* locus starts at ∼E10.5 and is almost complete at E13.5 (reactivated cells are shown in yellow; >85% of total cells). The XCR of PGCs progresses simultaneously with proliferation. The whole image of the embryo is shown on a modified diagram (top panel), based on Kaufman's Atlas of Mouse Development (Kaufman, 1992). The location of the GR analyzed is shown in red.

Our findings focusing on XCR in the *Pgk1* locus are summarized in Fig. 4. PGCs that reach the GR at E10.5 are scattered along the GR. PGCs proliferate in clusters from E11.5 onward, and XCR progresses according to embryonic development and PGC proliferation. PGC proliferation and XCR progression occur throughout the gonads without any particular spatial bias, and almost all PGCs complete XCR at E13.5.

## DISCUSSION

It is an important question as to whether PGCs undergo XCR in specific tissues before reaching the GR or whether it then progresses in specific regions in GR. Here, we found that XCR only occurred in PGCs that had reached the GR, and no particular structures were found in the surrounding somatic cells. Although a time lag in the arrival of individual PGCs might lead to uneven distribution, we found that PGCs were evenly scattered in the GR of E10.5 embryos. In the GR after E10.5, PGCs undergoing XCR also had no characteristic localization and were dispersed uniformly, indicating that there is no specific region of the GR that promotes XCR.

A characteristic feature of PGCs in the GR after E11.5 is that the cells form clusters and proliferate. At the same time, the number of cells undergoing XCR also increases gradually. This observation suggested a relationship between the XCR and DNA demethylation mechanisms in the development of PGCs. DNA demethylation in PGCs proceeds through two phases reviewed in Messerschmidt et al., 2014 and Zeng and Chen, 2019. The first phase is mainly passive, resulting in global demethylation (E8.5–E9.5). The second phase, which affects specific loci including inactivated X-linked genes, is believed to be initiated by ten-eleven translocation (TET) enzyme-mediated oxidation of 5-methylcytosine, followed by passive dilution of oxidized derivatives through cell division (E9.5–E13.5). The XCR pattern observed here was almost consistent with the timing of the reported second phase of DNA demethylation, supporting the role of active demethylation in XCR. Further analysis of 5-hydroxymethylcytosine on X chromosomes is required to clarify the role of TET enzyme-mediated active demethylation during XCR.

In the case of XCR in PGCs, we have demonstrated successfully that the timing of XCR differs depending on the gene locus using the Momiji system. We found that *Pgk1* undergoes XCR earlier than *Hprt* in PGCs. This is consistent with the order seen in the ICM during implantation, where *Pgk1* is classified as ‘late reactivated’ and *Hprt* as ‘very late reactivated’ (Borensztein et al., 2017). By contrast, during the establishment of induced pluripotent stem cells (iPSCs) *Hprt* undergoes XCR earlier than does *Pgk1* (Janiszewski et al., 2019). Reprogramming of iPSCs is an artificial response triggered by the introduction of reprogramming factors *in vitro*, suggesting that the kinetics of reprogramming in iPSCs might differ from those *in vivo*. The timing of X-linked gene activation using allele-specific RNA-sequencing in PGCs will help us to discriminate the characteristics of XCR between *in vivo* and *in vitro* situations.

Next, we focused on the XCR events that occur *in vivo* in the ICM and PGCs. The completion of XCR in PGCs is considered to involve a multistep process. Based on reporter gene expression in the Momiji system, derepression of the *Pgk1* locus began at ∼E10.5, but derepression of the *Hprt* locus was still incomplete at E13.5, indicating that XCR took more than 3 days. This is consistent with the results of expression analysis for individual X-linked endogenous transcripts using RT–PCR for DNA polymorphisms (Sugimoto and Abe, 2007). Here, the duration of XCR in PGCs was considerably longer than that in the ICM, where XCR takes about 1–2 days (E3.5–E5.5 by the Momiji system) (Kobayashi et al., 2016). One possible explanation for this difference might be the different repressive mechanisms of the two XCI states reprogrammed in each cell: imprinted XCI (iXCI) in the ICM, and random rXCI in PGCs. iXCI shares some mechanisms in common with rXCI, but the major difference is DNA methylation that is not present in iXCI. PGCs require the reprogramming of rXCI, and the erasure of epigenetic memories, including DNA methylation, might take longer. Differences in XCR caused by differences in the X chromosome regions (*Pgk1* and *Hprt*) and cell types (ICM and PGCs) are likely to be important clues for understanding the regulatory mechanism of XCI *in vivo*.

Recently, the expression analysis of X-linked transcripts of human PGCs was performed by single-cell RNA-seq, and it was reported that a gene dosage compensation mechanism called X chromosome dampening (XCD) acts when female PGCs undergo XCR (Chitiashvili et al., 2020). XCD equalizes the expression of X-linked genes between male and female embryos by reducing the amount of transcripts from each X chromosome in the female PGCs. It will be valuable in future studies to examine whether the expression level of X-linked transcripts is equalized between male and female mouse embryos by XCD when PGC undergoes XCR, to illuminate dosage compensation and the evolution of these mechanisms in different species.

Here, we succeeded in detecting XCR in PGCs using the Momiji reporter gene system and found that there are no specific XCR-promoting areas in the GR, but XCR proceeds rather ubiquitously while the PGCs proliferate as clusters. Thus, it is clear that the Momiji system can be used to detect XCR in all cells for which it has been reported, such as the ICM and embryonic stem cells (Kobayashi et al., 2016), PGCs in this paper, and iPSCs (submitted), indicating that this system has proved to be a very effective method for detecting XCR *in vivo* and *in vitro*.

## MATERIALS AND METHODS

### Animals

The Momiji mice used in this study were generated for previous studies (Kobayashi, 2018; Kobayashi et al., 2016). Here, four lines of Momiji mice were used, in which eGFP and mCherry fluorescent protein reporter cassettes (CAG-mCherry-NLS and CAG-eGFP-NLS) fused with a nuclear localization signal (NLS) were inserted into the *Hprt* and *Pgk1* loci. These mice can be obtained from the RIKEN BioResource Center (accession numbers: RBRC09532, RBRC09533, RBRC09535 and RBRC09536). Embryos obtained from crosses between female mice expressing eGFP and male mice expressing mCherry, or vice versa, were used. The Momiji mice were maintained by crossing with B6D2F1/Jcl mice (CLEA Japan, Inc., Tokyo, Japan). Eleven- to 34-week-old mice were used in mating experiments, and all efforts were made to minimize suffering.

### Cryosectioning

Samples to be sectioned were fixed overnight in 4% paraformaldehyde, incubated for over 4 h in 10% sucrose in phosphate-buffered saline (PBS), and then placed into 25% sucrose in PBS overnight. These steps were performed at 4°C. The samples were embedded in O.C.T. compound (Sakura Finetek Japan Co., Ltd., Tokyo, Japan) in cryomolds and frozen in liquid nitrogen.

### Immunohistochemistry

Frozen sections (5 µm) were washed three times with PBS every 5 min, permeabilized with PBS containing 0.2% Triton X-100 for 8 min on ice, and washed with PBS containing 0.1% Tween 20 (PBST) for 5 min. Blocking treatment with PBS containing 1% Roche blocking reagent (Roche, Basel, Switzerland) was performed at room temperature for 1 h. The sections were incubated using anti-Oct3/4 (catalogue number 09-0023, 1:100 dilution; Stemgent Inc., Cambridge, MA, USA) or anti-DDX4/MVH (ab13840, 1:500 dilution; Abcam, Cambridge, UK). Fluorescence immunohistochemical detection was performed using a donkey anti-rabbit IgG H&L (Alexa Fluor 647) secondary antibody (ab150075, 1:300 dilution; Abcam) and DAPI (340-07971, 1:1000 dilution; Dojindo, Kumamoto, Japan). After treatment with the primary or secondary antibodies, they were washed three times with PBST for 15 min. The sections were sealed with ProLong Diamond Antifade Mountant (Thermo Fisher Scientific, Inc., Waltham, MA, USA).

### Fluorescence Imaging and quantification

Observation and quantification of signals was performed as described (Kobayashi, 2018). Fluorescence images were acquired using an Olympus FLUOVIEW FV1000 confocal laser scanning microscope, and quantification of the fluorescence signals was performed using dedicated FLUOVIEW software (Olympus Corp., Tokyo, Japan). To calculate the number of PGCs in the GR, it was not practicable to count all cells in all slides, so we estimated the count from three selected sagittal sections centered on the longest axis of the gonad. This ‘index of number of PGCs’ was the mean number of Oct3/4-positive or Mvh-positive PGCs counted on these sections.

### Ethics statement

This study was carried out in strict accordance with the Japanese National Institute of Advanced Industrial Science and Technology guidelines for life science experiments (accreditation Nos. K2016-0035 and A2020-0306). Animal experiments were performed in accordance with the Guidelines for Proper Conduct of Animal Experiments stipulated by the Science Council of Japan.

## Supplementary Material

Supplementary information
