# Supplementary material for: Visualization of X chromosome reactivation in mouse primordial germ cells in vivo
Source: Biol Open. 2021 Apr 29;10(4):bio058602. doi: 10.1242/bio.058602 (PMC8096617; doi:10.1242/bio.058602)
Supplement: Supplementary information [file biolopen-10-058602-s1.pdf]

**Fig. S1**

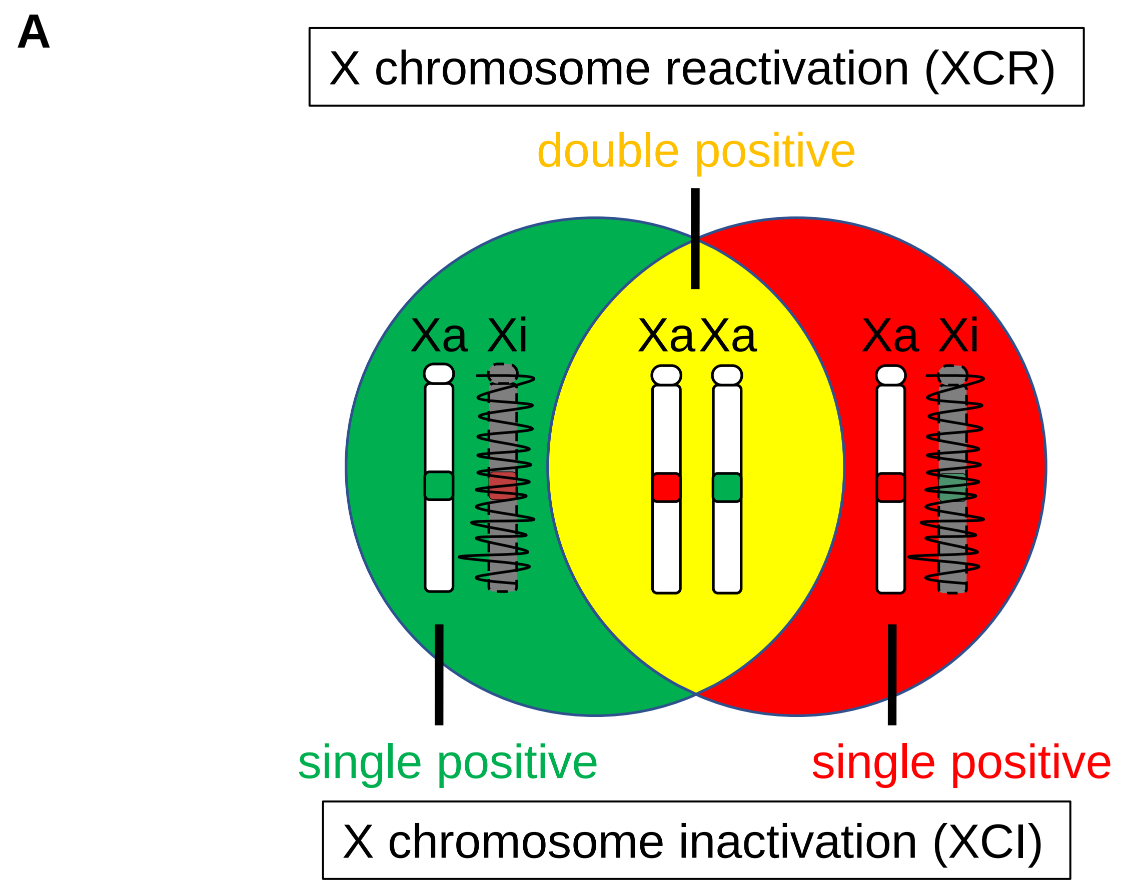


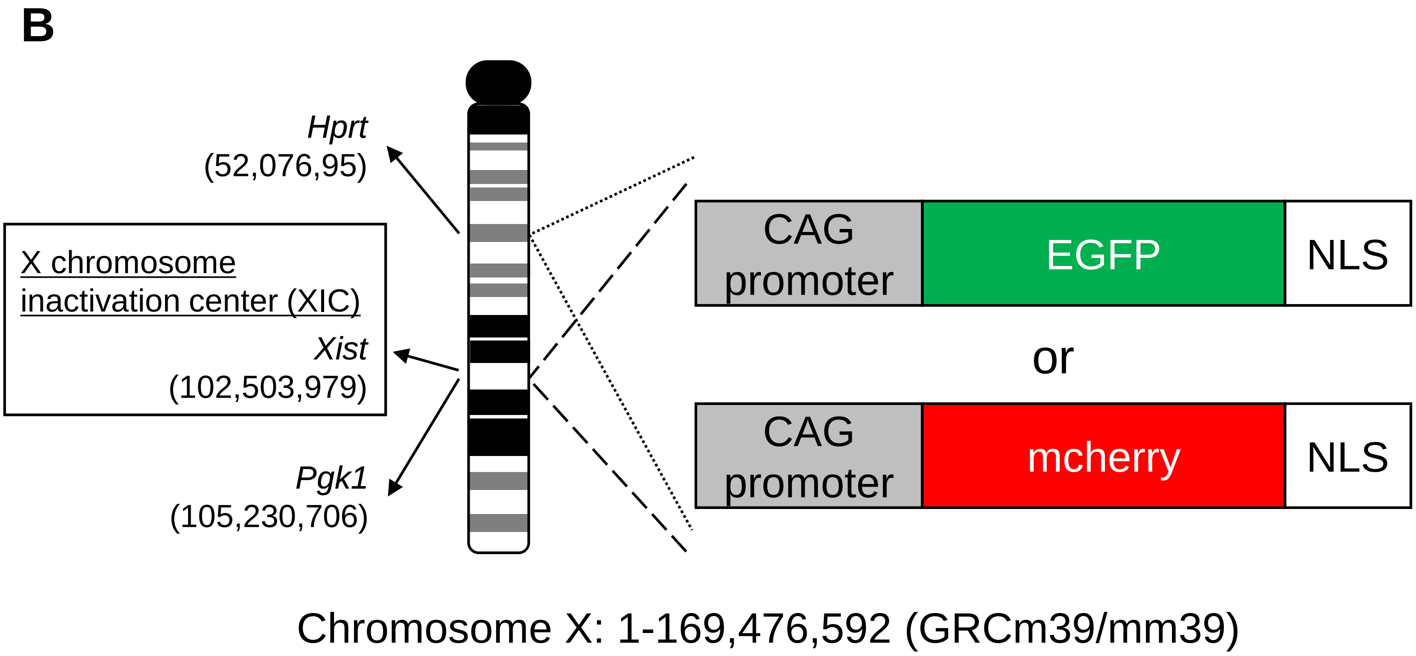


| locus | EGFP (green) | mcherry (red) |
|---|---|---|
| *Hprt* | *Hprt*-Green (HG) | *Hprt*-Red (HR) |
| *Pgk1* | *Pgk1*-Green (PG) | *Pgk1*-Red (PR) |

Biology Open • Supplementary information

**Fig. S1. Concept of the Momiji system.** (A) Two separate fluorescent protein reporter genes (*mCherry* and *EGFP*) were inserted into mouse X chromosomes. In this system, single positive green or red signals indicate active individual X chromosomes. Double positive signals indicate XCI. Xa, active X chromosome; Xi, inactive X chromosome. (B) Two gene loci (*Hprt* and *Pgk1*) of X chromosomes were used for the insertion of fluorescent reporter genes. NLS: nuclear localization signal.

**Fig. S2**

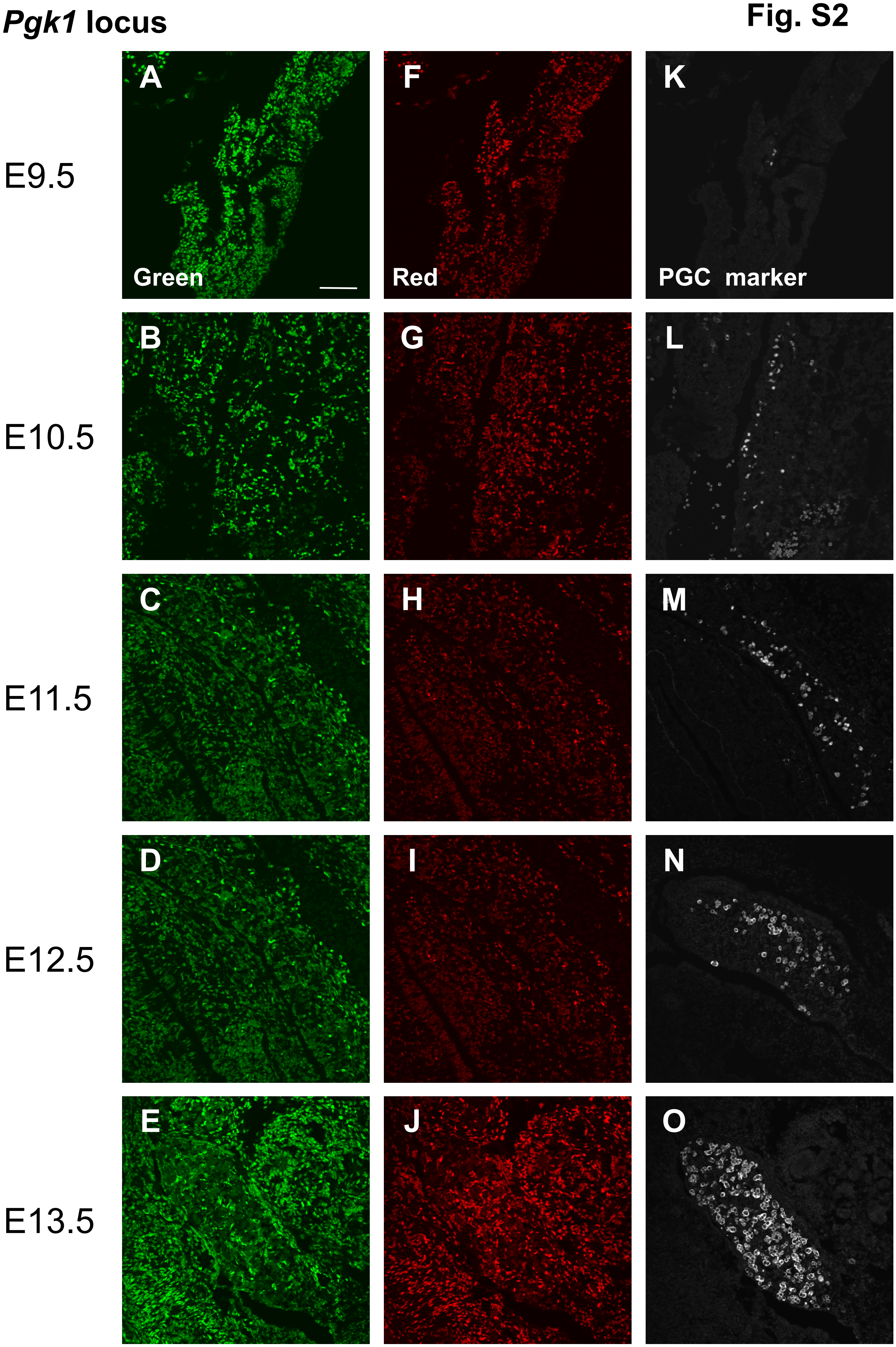


Biology Open • Supplementary information

**Fig. S2. Original images of Fig. 1A–E.** (A–E) Images for eGFP (green). (F–J) Images for mCherry (red). (K–O) Immunostaining images for PGC markers (Oct3/4 for E9.5- E11.5, and Mvh for E12.5 and E13.5). Developmental stages are indicated on the left side of the panels. Scale bar: 100 μm.

Biology Open (2021): doi:10.1242/bio.058602 Supplementary information

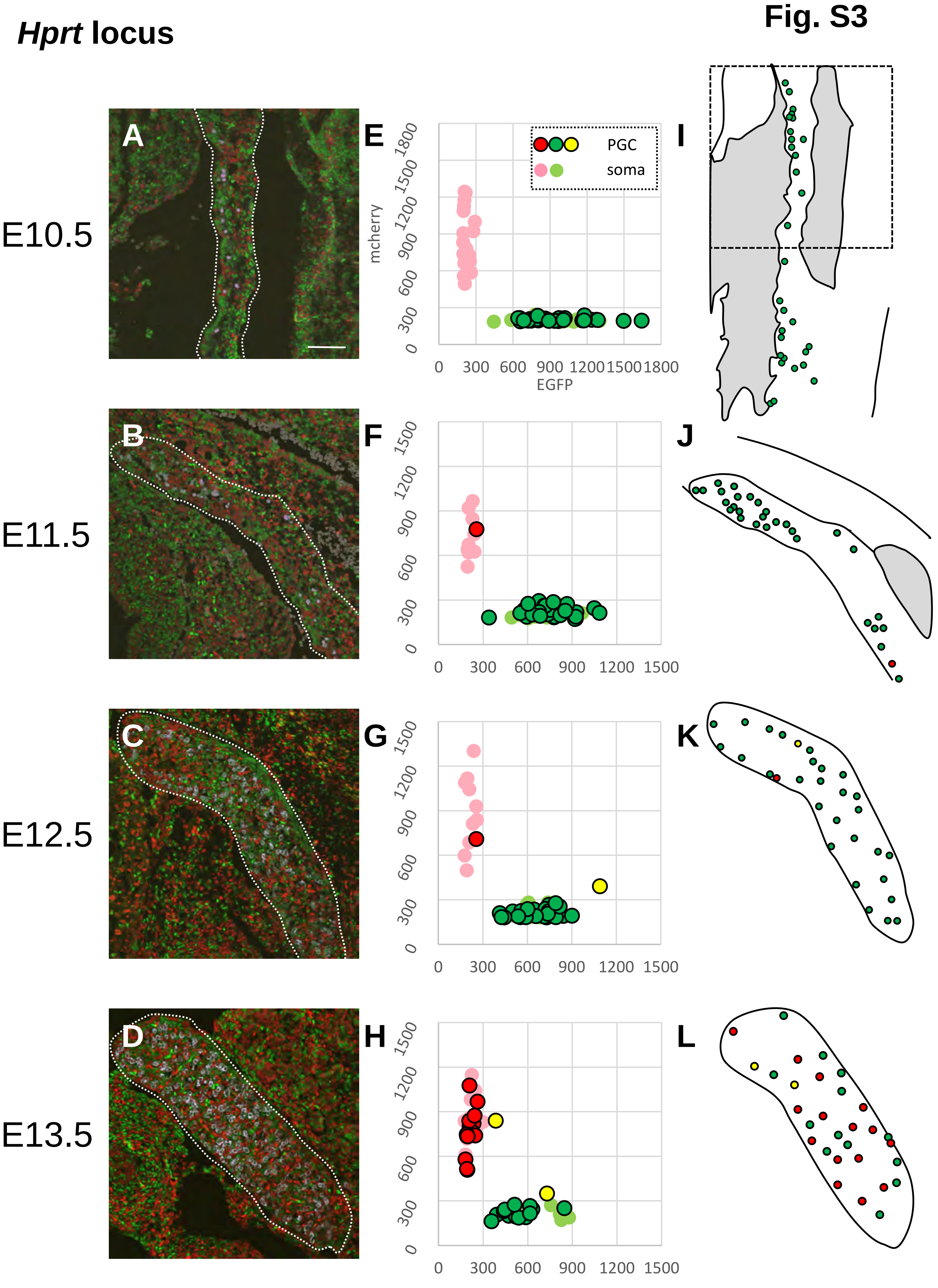


Biology Open • Supplementary information

**Fig. S3. Observation of X chromosome reactivation of the *Hprt* locus in PGCs at each developmental stage.** (A–D) Merged images of eGFP (green), mCherry (red), and immunostaining with PGC markers (Oct3/4 for E10.5 and E11.5, and Mvh for E12.5 and E13.5) (white) are shown. The GR is indicated by dotted lines. Scale bars: 100 μm. (E–H) Detection of XCR by quantitative analysis of fluorescence intensity in PGCs. Each spot corresponds to one cell. Ten positive somatic cells in light red or light green were plotted. PGC marker-positive cells are shown as circles outlined in black, and cells with both red and green signal intensities >300 are shown as yellow. Prior to E10.5, all PGCs in a slide are plotted. Because the number of PGCs becomes enormous after E11.5, the number in these plots was limited to a maximum of 30. (I–L) Schematic representation of the XCI (red or green) or XCR (yellow) of PGCs located in the genital ridge. The boxed region in I indicates the area photographed in A. The positions of PGCs whose signal intensities were measured in E-H are shown in these images. Developmental stages are indicated on the left side of the panels. For each stage, at least three embryos were analyzed, and a representative one is shown here.

Fig. S4

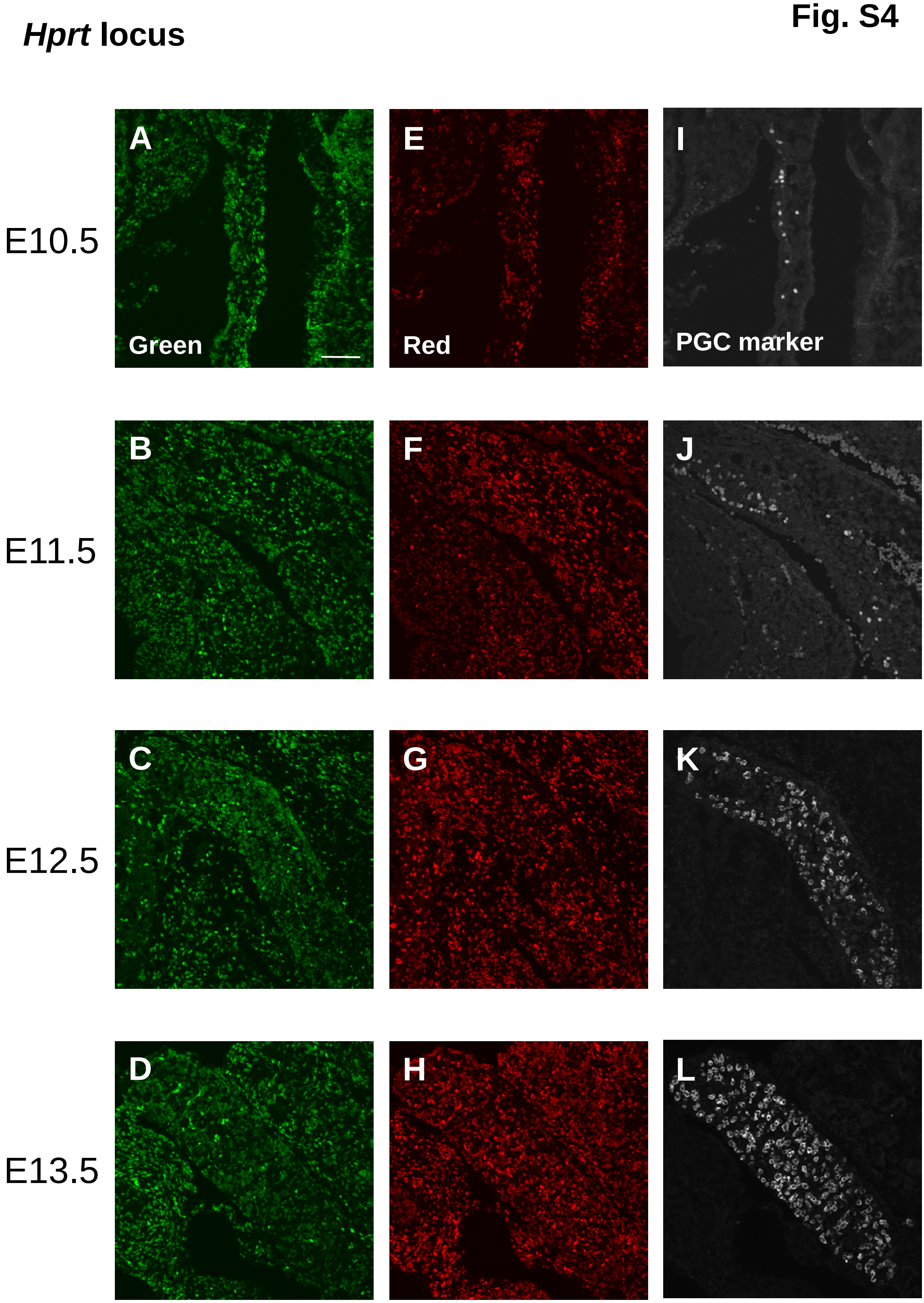
*Hprt* locus
E10.5
E11.5
E12.5
E13.5
A
Green
E
Red
I
PGC marker
B
F
J
C
G
K
D
H
L

**Fig. S4. Original images of Fig. S3A–D.** (A–D) Images for eGFP (green). (E–H) Images for mCherry (red). (I–L) Immunostaining images for PGC markers (Oct3/4 for E10.5 and E11.5, and Mvh for E12.5 and E13.5). Developmental stages are indicated on the left side of the panels. Scale bar: 100 μm.

| cross(♀ × ♂) | embrionic stage | PGC makers used for detection | number of PGCs (avg. of 3 slides) | average number of PGCs | SD | XCR rate (%) | avarage of XCR rate (%) | SD |
|---|---|---|---|---|---|---|---|---|
| PR×PG | E10.5 | Oct | 44 | 24.4 | 15.8 | 15.15 | 24.8 | 20.2 |
| PR×PG | | Oct | 24 | | | 52.86 | | |
| PR×PG | | Oct | 5.33 | | | 6.25 | | |
| PR×PG | E11.5 | Oct | 48.33 | 83.8 | 26.6 | 38.89 | 32.6 | 6.7 |
| PR×PG | | Oct | 112.33 | | | 23.33 | | |
| PR×PG | | Oct | 90.67 | | | 35.56 | | |
| PR×PG | E12.5 | MVH | 116.33 | 123.8 | 5.6 | 68.89 | 69.8 | 10.2 |
| PR×PG | | MVH | 125 | | | 57.78 | | |
| PR×PG | | MVH | 130 | | | 82.67 | | |
| PR×PG | E13.5 | MVH | 336 | 339.0 | 20.9 | 94.44 | 86.7 | 11.0 |
| PR×PG | | MVH | 366 | | | 71.11 | | |
| PR×PG | | MVH | 315 | | | 94.44 | | |

**TableS1, Index of number of PGCs and percentage of XCR-initiated PGCs (*Pgk1* locus)**

| cross(♀ × ♂) | embrionic stage | PGC makers used for detection | number of PGCs (avg. of 3 slides) | average number of PGCs | SD | XCR rate (%) | avarage of XCR rate (%) | SD |
|---|---|---|---|---|---|---|---|---|
| HG×HR | E10.5 | Oct | 23.0 | 17.5 | 5.5 | 1.45 | 2.1 | 0.7 |
| HR×HG | | Oct | 12.0 | | | 2.78 | | |
| HG×HR | E11.5 | Oct | 91.0 | 73.0 | 13.0 | 1.67 | 0.6 | 0.8 |
| HR×HG | | Oct | 67.3 | | | 0 | | |
| HR×HG | | Oct | 60.7 | | | 0 | | |
| HG×HR | E12.5 | MVH | 183.0 | 211.0 | 39.8 | 8.89 | 3.7 | 3.8 |
| HG×HR | | MVH | 267.3 | | | 0 | | |
| HG×HR | | MVH | 182.7 | | | 2.22 | | |
| HG×HR | E13.5 | MVH | 292.0 | 353.1 | 57.1 | 18.89 | 11.5 | 5.3 |
| HR×HG | | MVH | 338.0 | | | 6.67 | | |
| HR×HG | | MVH | 429.3 | | | 8.89 | | |
| HG×HR | neonate | MVH | 392.0 | 388.0 | 6.9 | 90 | 92.2 | 1.9 |
| HG×HR | | MVH | 380.0 | | | 93.33 | | |
| HG×HR | | MVH | 392.0 | | | 93.33 | | |

**TableS2, Index of number of PGCs and percentage of XCR-initiated PGCs (*Hprt* locus)**

Biology Open • Supplementary information
